# Integrated multi-omics analysis of dampness-heat gout reveals diagnostic biomarkers and therapeutic targets

**DOI:** 10.3389/fimmu.2026.1677920

**Published:** 2026-02-06

**Authors:** Le Yang, Ye Sun, Chuanning Li, Hui Sun, Shuyun Wei, Fangjie Zhu, Wenkai Wang, Runyue Huang, Xin Sun, Maojie Wang, Guangli Yan, Xijun Wang

**Affiliations:** 1 State Key Laboratory of Dampness Syndrome of Chinese Medicine, The Second Affiliated Hospital of Guangzhou University of Chinese Medicine, Guangzhou, China; 2 National Chinmedomics Research Center, National Traditional Chinese Medicine (TCM) Key Laboratory of Serum Pharmacochemistry, Metabolomics Laboratory, Department of Pharmaceutical Analysis, Heilongjiang University of Chinese Medicine, Harbin, China; 3 State Key Laboratory of Quality Research in Chinese Medicine, Macau University of Science and Technology, Macao, Macao SAR, China

**Keywords:** biomarkers, dampness-heat gout, diagnosis and treatment model, multi-omics, TCM

## Abstract

Damp-heat gout (DHG) is a highly certified type of disease integrated with syndrome in TCM. The ambiguity of its pathomechanism and the lack of quantifiable indicators limit its clinical accurate diagnosis and treatment. This study aimed to elucidate the pathological mechanism of DHG and establish a symptom-centered diagnostic and therapeutic model. We recruited 136 participants, comprising healthy controls (HCs) and DHG patients. Serum metabolomics and proteomics analyses were performed to screen common pathways. Based on the biological significance of these common pathways, a symptom-pathway correlation network was constructed to clarify the pathological mechanisms driving DHG occurrence and progression. Enrichment scores and correlations with key DHG symptoms were used to identify critical pathways. Differential metabolites and proteins associated with these critical pathways served to establish a multi-index diagnostic model and identify potential therapeutic protein targets. Integrated metabolomic and proteomic analyses revealed 21 common pathways associated with DHG. Four crucial pathways, such as Bile secretion, Cholesterol metabolism, Purine metabolism, Arachidonic acid metabolism, were exhibited significant correlations with core DHG symptoms. Furthermore, six pathway-related biomarkers were identified: Hypoxanthine, Prostaglandin E2, Uric acid, Deoxycholic acid, Taurochenodeoxycholic acid, and Bilirubin. The combined diagnostic efficacy of these biomarkers was optimal (discovery cohort: AUC = 0.987; validation cohort: AUC = 0.997). Six protein targets were identified from the crucial pathways, including ATP1A1, APRT, ANGPTL4, GLUT1, PTGES3 and LIPA. This study establishes a symptom-centered diagnostic and therapeutic model for DHG utilizing the identified biomarkers and clarifies the involvement of critical metabolic pathways in DHG pathogenesis, providing novel targets for improved clinical diagnosis and therapy.

## 1 Introduction

Gout is the most common form of inflammatory arthritis worldwide; it is caused by the deposition of sodium urate crystals in joints and affects about 3.9% of the global population (1–4). Long-term recurrent episodes can lead to permanent joint deformation and severe damage to renal function (5, 6) and may be attributed to changes in diet and lifestyle (1, 7, 8). Although all gout patients present manifestations of joint swelling and heat pain, the physical state of patients after suffering from the disease is different, and thus, the treatment plan should also be different. Traditional Chinese medicine (TCM) divides acute gouty arthritis into multiple syndrome types based on clinical manifestations (damp-heat syndrome, phlegm block syndrome, stasis and heat block syndrome, liver and kidney Yin deficiency syndrome, etc.) (9). Damp-heat gout (DHG) is the most common disease associated with TCM syndrome and is characterized by sudden red swelling and heat pain in the small joints of the lower limb, fever and heaviness, agitation, yellow urine, red tongue, yellow and greasy coatings, and slippery and rapid pulses (10–12). TCM suggests that the blockage of “dampness” and “heating” in joints leads to the occurrence of DHG (13), and the increase in uric acid levels and the redness and swelling of joints are considered manifestations of dampness-heating. The method of removing “dampness heat” in the treatment of DHG was also found to be effective (14). The diagnosis of DHG mostly depends on the personal experience of clinicians, and few quantifiable and stable indicators are available. This often delays the diagnosis, especially in the early stage, leading to recurrent joint inflammation. Therefore, novel predictive biomarkers are needed for the prompt diagnosis of DHG.

Although international gout research has made significant advances in understanding uric acid metabolism and inflammatory pathways (15–17) and many researchers have started investigating associations between TCM dampness–heat syndrome and molecular indicators (such as metabolic and microbial dysbiosis) (18, 19), a systematic and specific composite biomarker panel for DHG needs to be identified. While traditional single indicators such as serum uric acid or monosodium urate crystals in synovial fluid have diagnostic value at the disease level, they fail to accurately capture the complex pathophysiology of dampness–heat syndrome, and their diagnostic performance is often compromised in different sample populations. This gap necessitates a breakthrough using systems biology approaches. Multi-omics technologies, as core tools of systems biology, enable large-scale parallel analysis of molecular populations (e.g., proteins and metabolites) within organisms (20–22). They comprehensively capture multilayered information, including metabolic disturbances and protein–metabolite interaction networks, and use such information to construct multidimensional molecular maps of diseases. Their powerful network association analysis capabilities reveal molecular interactions and core regulatory pathways, offering a material basis for elucidating TCM pathogenesis.

In this study, we used metabolomics combined with proteomics technology to focus on the key biological pathways of DHG and link the identified core molecules and pathways with the core symptoms of dampness–heat syndrome. Through omics data integration and pattern recognition methods such as machine learning, a combined biomarker group that can not only reflect disease syndrome manifestations but also distinguish patients with DHG from healthy people can be identified by simultaneously constructing a DHG multi-index diagnostic model, greatly improving the precision and reliability of diagnosis. We also revealed therapeutic targets corresponding to the biomarker group and the main symptoms, providing a theoretical basis for the personalized treatment of DHG. This study provides a reference for the clinical diagnosis and treatment of DHG and offers a new paradigm for modern research on TCM-related disease-syndrome combined diseases.

## 2 Materials and methods

### 2.1 Study participants

This study enrolled 86 male patients diagnosed with DHG from Guangdong Provincial Hospital of Chinese Medicine between 2020 and 2022, along with 50 age-matched healthy controls (HCs) recruited from Guangdong Eleventh People’s Hospital in 2022. DHG patients met the following inclusion criteria: (a) fulfillment of the 2015 ACR/EULAR classification criteria for gouty arthritis; (b) compliance with diagnostic standards for DHG outlined in the Guideline for Diagnosis and Treatment of Gout and Hyperuricemia with Integrated Traditional Chinese and Western Medicine (12); (c) age 18–70 years; (d) exclusion of individuals with severe cardiovascular, cerebrovascular, pulmonary, hepatic, renal, hematologic malignancies, psychiatric disorders, or secondary gout induced by underlying conditions or medications. HCs were included based on (a) self-reported absence of significant medical conditions and (b) normal physical examination findings within the same age range.

**Figure 1:**
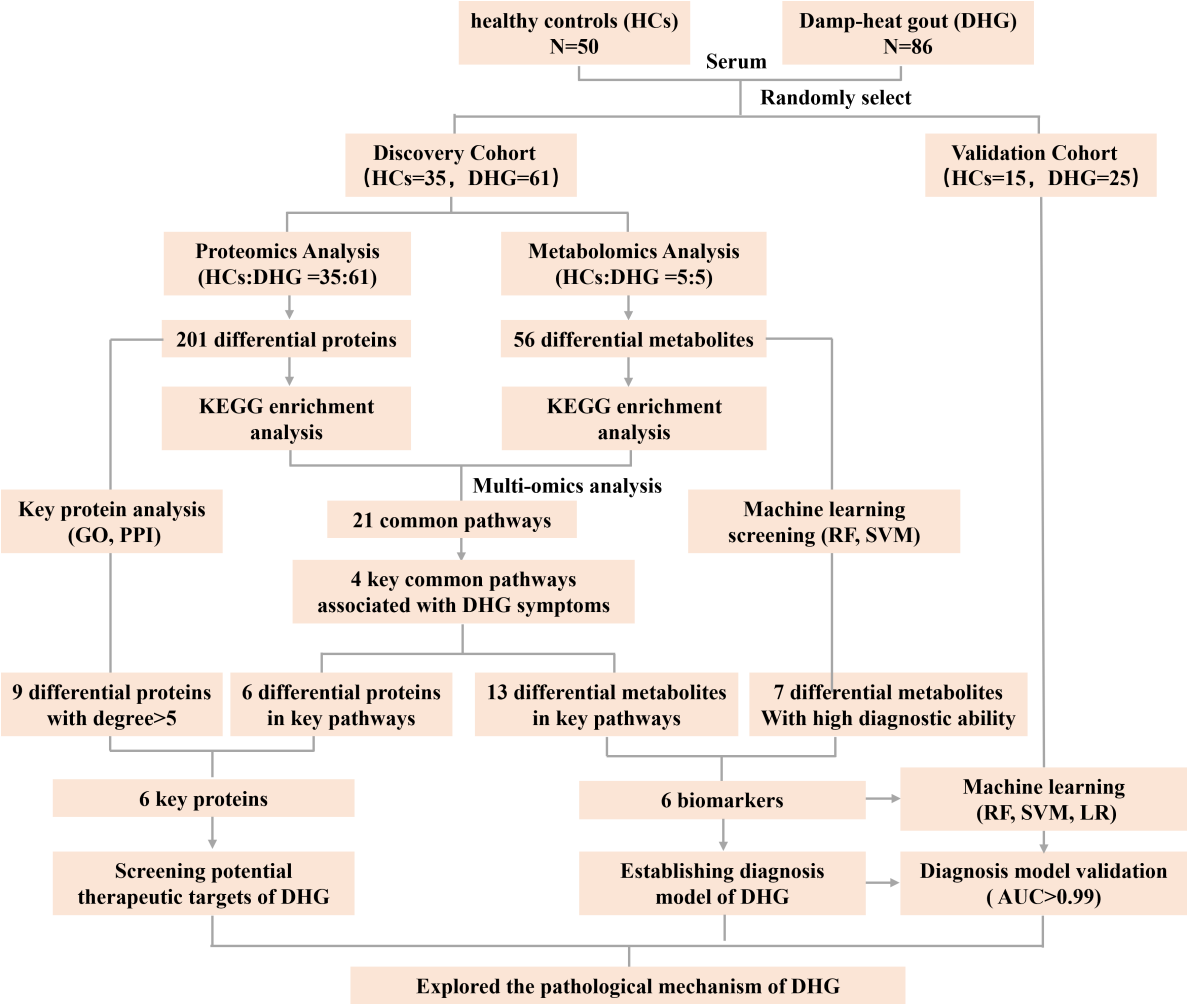
Study design. A total of 136 subjects were enrolled in present study, included both discovery cohort and validation cohort. HCs, Healthy controls, DHG, damp-heat gout, GO, Gene Ontology, PPI, protein-protein interaction, SVM, support vector machine, LR, logistic regression, AUC, area under curve.

Using computer-generated random number tables, we allocated participants into discovery (60 DHG patients and 35 HCs) and validation cohorts (25 DHG patients and 15 HCs). The discovery cohort served for initial biomarker identification, while the validation cohort was utilized for subsequent optimization and verification of findings (**Figure 1**). Importantly, no significant age differences were observed between DHG patients and HCs within either cohort (**Table 1**), ensuring proper case-control matching.

**Table 1:** Baseline characteristics of the participants in discovery cohorts and validation cohorts.

Variables	Training cohort		Validation cohort	
Parameter	HC (n=35)	DHG (n=61)	HC (n=15)	DHG (n=25)
Age (years)	44.66 ± 8.38	40.98 ± 12.55	43.53 ± 11.34	40.36 ± 12.98
BMI (kg/m2)	23.72 ± 1.69	25.29 ± 2.94***	22.54 ± 1.81	25.54 ± 2.20***
GLU (mmol/L)	5.16 ± 0.61	5.63 ± 2.72	5.21 ± 0.65	5.22 ± 0.52
UA (μmol/L)	354.30 ± 48.65	467.20 ± 158.40***	344.50 ± 67.90	426.70 ± 147.90***
TC (mmol/L)	4.52 ± 0.47	4.93 ± 0.85***	4.21 ± 0.64	4.86 ± 0.92***
TG (mmol/L)	1.04 ± 0.29	2.30 ± 2.01**	1.05 ± 0.34	2.08 ± 1.35**
HDL-C (mmol/L)	1.43 ± 0.27	1.21 ± 0.44*	1.43 ± 0.27	1.21 ± 0.44*
LDL-C (mmol/L)	2.78 ± 0.52	3.26 ± 0.88***	2.52 ± 0.47	3.19 ± 0.73***

Comparison between DHG group and HCS group: *P<0.05, **P<0.01, ***P<0.001.

### 2.2 Clinical characteristics and sample collection

All participant anthropometric data, including age, BMI, blood glucose, uric acid, and other biochemical parameters. Participants provided fasting venous blood samples after an overnight fast and without any medication. The blood samples were allowed to clot at room temperature for 30 minutes, followed by centrifugation at 3000 rpm for 10 minutes to obtain serum.

### 2.3 Metabolomic analysis

#### 2.3.1 Sample preparation

50 μL of each serum sample was mixed with 200 μL of methanol and shaken for 1 minute. The mixture was then left to stand at 4°C for 20 minutes, followed by centrifugation at 13,000 rpm for 15 minutes. The supernatant was filtered through a 0.22 μm membrane for subsequent UPLC-MS analysis. A quality control (QC) sample was prepared by pooling all serum samples aliquots.

#### 2.3.2 UPLC-MS analysis

The metabolomics analysis was performed using the UPLC (Ultimate 3000, Thermo Scientific) and Orbitrap analysis platform (Q-Exactive Focus, Thermo Scientific). Chromatographic separation was performed on Waters 2.1 mm × 100 mm ACQUITY UPLC HSS T3 1.8µm chromatographic column (Waters, USA) at 35°C. The mobile phase consisted of acetonitrile (A) and 0.1% formic acid in water (B). The UPLC gradient program were: 0 ~ 1.0 min, 95% ~ 75% B; 1.0 ~ 2.0 min, 75% ~ 40% B; 2.0 ~ 7.5 min, 40% ~ 10% B; 7.5 ~ 10.5 min, 10% ~ 1% B; 10.5 ~ 12.5 min, 1% B; 12.5 ~ 13.0 min, 1% ~ 95% B; 13.0 ~ 15.0 min, 1% B. The flow rate was 0.30 ml/min and the inject sample volume was 3 μL. After separation, mass spectrometry was performed on a Q-Exactive Focus. Heater temperature 320°C; Sheath gas flow rate: 40; Auxiliary gas flow rate: 15; Sweep gas flow rate: 1; Positive ion electrospray voltage: 3.5 KV, negative ion electrospray voltage: 3.2 KV; Capillary temperature: 350°C; S-Lens RF Level: 50. Scanning mode: Full Scan (m/z 80-1200), resolution 70,000.

QC sample was analyzed once every 10 test samples. The primary data files (formatted as.raw) were exported to Progenesis QI software (Nonlinear Dynamics, version: 2.0) for data pre-treatment, including peak alignment, peak picking, deconvolution, and identification.

### 2.4 Statistical analysis

Explored overall metabolic changes between the groups using principal component analysis (PCA) and hierarchical clustering analysis. Employed orthogonal partial least squares discriminant analysis (OPLS-DA) to maximize inter-group metabolic variations and evaluated the model’s discriminative power with 100 permutations to guard against overfitting. The matrix result was reduced by replacing the missing values, and data with >20% missing values were removed. Univariate analysis was performed with Wilcoxon Mann-Whitney U test, the p values were adjusted with false discovery rate (FDR) correction. Then, the ions which conform to VIP > 0.5, P < 0.05, log2(FC)>1.5 were selected. These ions were deemed to have a large contribution rate to the change of the metabolic profile. The qualifying ions were screened for endogenous metabolites through the Human Metabolome Database (HMDB) (http://www.hmdb.ca/). The mass spectrometric data collected in FullMS-ddMS2 mode, and the structure of differentially expressed metabolites was confirmed by combining the compound excimer ions, MS cleavage pathways, and some reference substances. Pathway enrichment analysis was conducted using The R package through the MetaboAnalyst web tool (https://www.metaboanalyst.ca/).

### 2.5 Proteomic analysis

#### 2.5.1 Sample preprocessing

Five serum samples were randomly selected from each of the DHG patients and HCs. In total, 40 μL of 2.5% magnetic bead suspension was removed. After cleaning, the magnetic beads were resuspended in 100 μL of wash buffer. Then, 100 μL of serum was added, and the mixture was incubated at 37°C and mixed evenly for 1 h. The supernatant was discarded by magnetic separation, and the mixture was washed with a wash buffer three times to obtain enriched serum protein. In total, 40 μL of the reductive alkylation and enzymatic hydrolysis system was added to the protein-enriched magnetic beads. After blowing and mixing, 1.2 μL of reductive alkylation reagent was added. After mixing, the mixture was heated at 95°C for 5 min. After cooling to room temperature, 2 μL of equilibrium solution and 5 μL of enzymolysis solution were added, and the mixture was mixed well and subjected to enzymolysis at 37°C for 2 h. After the termination reagent was added, the mixture was centrifuged at 20,000 × g for 1 min, the supernatant was removed, a C18 column was used for peptide desalting, and the sample was concentrated using a vacuum freeze-centrifugation concentrator.

#### 2.5.2 LC-MS/MS high-resolution mass spectrometry detection

Peptide quantification was performed using a NanoDrop spectrophotometry system. Prior to mass spectrometric analysis, experimental samples were spiked with IRT internal standard at a 1:20 volume ratio (standard: sample). The chromatographic separation employed a DIA-enabled LC system configured with: 400 nL/min flow rate through a 15 cm × 75 μm ID C18 analytical column (IonOpticks, 1.6 μm beads), using mobile phase A (0.1% formic acid in water) and phase B (0.1% formic acid in acetonitrile). A complete gradient profile is documented in **Supplementary Table 1**.

Mass spectrometry acquisition parameters were optimized as: electrospray voltage (1.4 kV), desolvation temperature (180°C) with 3.0 L/min nebulizing gas, full scan range 100–1700 m/z, ion mobility window 0.7-1.3 V·s/cm², and collisional energy ramp from 20 to 59 eV. Instrument control and data acquisition were performed using manufacturer-specified workflows.

#### 2.5.3 Data processing and analysis

The DIA raw data were imported into Spectronaut Pulsar 18.4 (Biognosys) software for spectrum matching, quantitative information extraction, and subsequent statistical analysis. Differentially expressed proteins were identified using selection criteria of P < 0.05 with fold change thresholds <0.5 (downregulation) or >2 (upregulation). Functional characterization of the detected proteins was achieved through multi-database annotation analysis, systematically interrogating Gene Ontology (GO) terms, KEGG pathways, and Pfam protein families to delineate biological functions. Homo sapiens was selected in the STRING database was used to analyze the protein-protein interaction (PPI) networks of the differential proteins.

### 2.6 Multi-omics integration of proteome and metabolome data

Differentially expressed metabolites and proteins were individually subjected to KEGG pathway enrichment analysis, and common intersecting pathways were screened. The diagnostic symptoms for DHG are based on the Guideline for Diagnosis and Treatment of Gout and Hyperuricemia with Integrated Traditional Chinese and Western Medicine (12). Relationships between these common pathways and the symptoms of DHG were determined through literature review to construct a metabolite/protein-common pathway-symptom relationship network. Diverse machine learning algorithms, including Support Vector Machine (SVM), Logistic Regression (LR), and Random Forest (RF), were used for analysis within the machine learning model. Receiver operating characteristic (ROC) curves were generated using Monte-Carlo cross-validation with balanced subsampling. The analysis was conducted using the R package MetaboAnalystR.

## 3 Result

### 3.1 Clinical characteristics of enrolled participants

Initially, a comparative analysis of clinical biochemical parameters between the DHG group and HCs was conducted in both the discovery and validation cohorts. Consistent findings were observed across both datasets: The DHG group showed no significant differences in age or blood glucose compared to the HCs group, while marked discrepancies were identified in BMI, UA (uric acid), TC (total cholesterol), TG (triglycerides), HDL-C (high-density lipoprotein cholesterol), and LDL-C (low-density lipoprotein cholesterol) levels (**Table 1**). These results suggest that patients with dampness-heat pattern gout commonly presented with obesity, hyperuricemia, and hyperlipemia. These abnormalities are closely related to systemic metabolic disorder.

### 3.2 Deoxycholic acid, hypoxanthine, prostaglandin E2, and other 53 substances were identified as differential metabolites of DHG

**Figure 2:**
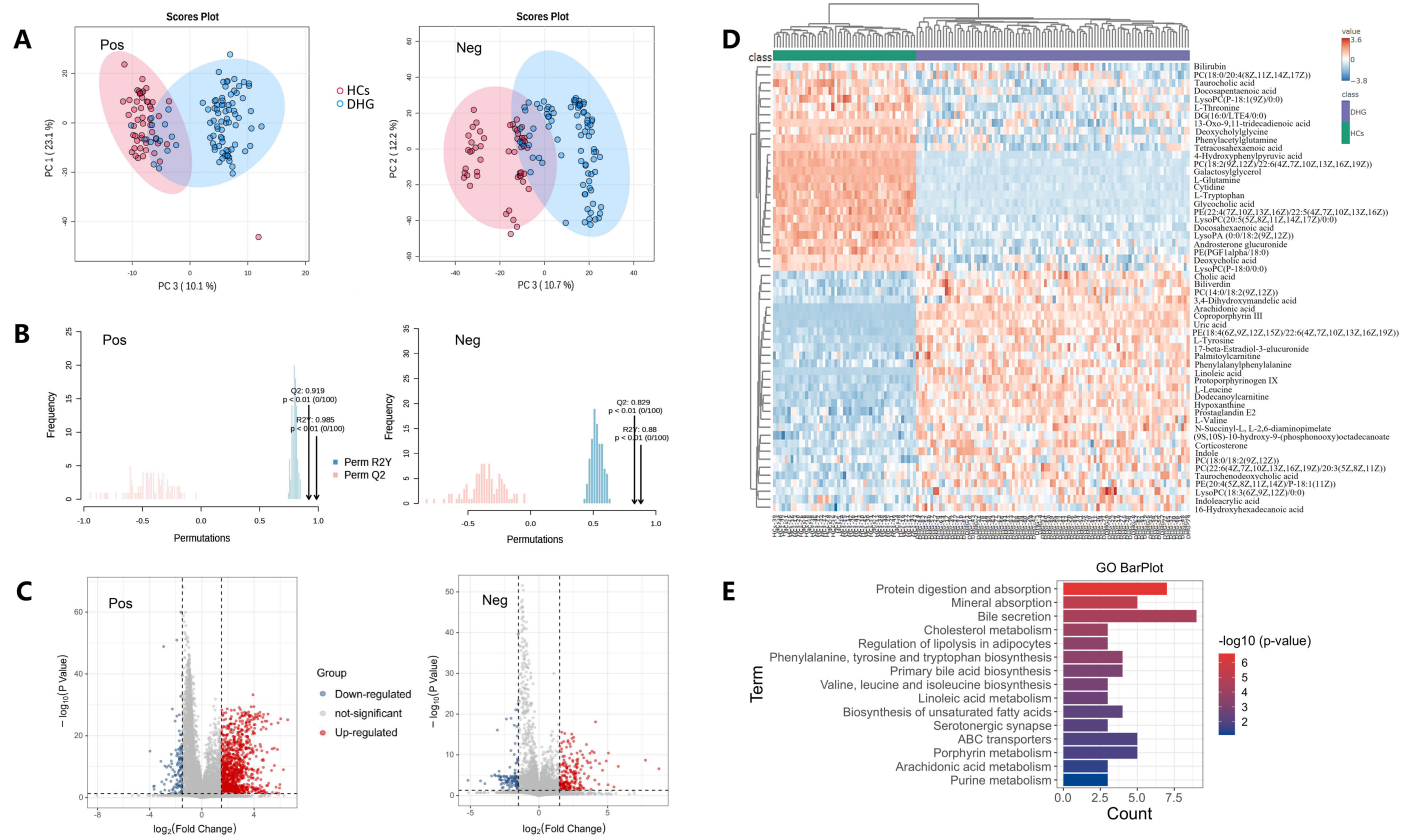
Screening and enrichment analysis of differential metabolite. **(A)** PCA scatter plots showed that the metabolic data of healthy controls and DHG patients had good separation characteristics. **(B)** The results of 100 alignment tests of positive and negative OPLS-DA show that the model is not overfitted. **(C)** Volcanic map of P value and fold change. **(D)** Heat map of 56 differential metabolites. **(E)** KEGG enrichment analysis map of 56 differential metabolites.

Metabolomics analysis was conducted on serum samples from two groups in discovery cohorts, resulting in a total of 27,233 metabolic features detected in both positive and negative ion modes. PCA was utilized for assessing the inherent metabolic variations and data quality of all features (**Figure 2A**). Notably, clear overall trends were observed, with a distinct separation between the damp-heat syndrome of gout and control groups. OPLS-DA demonstrated complete separation of the overall metabolic characteristics between the control and damp-heat syndrome of gout groups. We tested 100 permutations under both positive and negative ion modes. In terms of the positive ion mode, Q2 = 0.919, R2Y = 0.985, p < 0.01, while for the negative ion mode, Q2 = 0.829, R2Y = 0.88, p < 0.01. The results indicate that the model has been proven to be effective, without overfitting, and can be used for subsequent analysis (**Figure 2B**). Metabolites satisfying the criteria of VIP > 0.5, p < 0.05, and a FC > 1.5 were designated as candidate biomarker features (**Figure 2C**). These candidate features then underwent MS^2^ analysis, with the fragment information utilized for identification against the HMDB database. Finally, 56 substances such as deoxycholic acid, hypoxanthine and prostaglandin E2 were identified as differential metabolites of DHG. (**Table 2**). The difference in 56 metabolites content between the DHG and HCs can be clearly shown by heat map (**Figure 2D**). Subsequently, KEGG enrichment analysis was performed on the 56 different metabolites. The results revealed that Bile secretion, Protein digestion and absorption, Cholesterol metabolism and other 60 pathways were disturbed. (**Figure 2E**; **Supplementary Table 2**).

**Table 2:** Results of the identification of differential metabolites.

NO.	RT	Metabolite	m/z	Ion	Molecular	HMDB	KEGG	Trend
M1	0.86	L-Glutamine	145.0606	[M-H]^-^	C_5_H_10_N_2_O_3_	HMDB0000641	C00064	Down
M2	0.87	L-Threonine	120.0656	[M+H]^+^	C_4_H_9_NO_3_	HMDB0000167	C00188	Down
M3	0.9	L-Valine	118.0863	[M+H]^+^	C_5_H_11_NO_2_	HMDB0000883	C00183	Down
M4	0.93	Cytidine	242.0793	M-H	C_9_H_13_N_3_O_5_	HMDB0000089	C00475	Down
M5	0.95	Hypoxanthine	137.0459	[M+H]^+^	C_5_H_4_N_4_O	HMDB0000157	C00262	Up
M6	0.95	Uric acid	169.0357	[M+H]^+^	C_5_H_4_N_4_O_3_	HMDB0000289	C00366	Up
M7	1.74	L-Tyrosine	180.0656	M-H	C_9_H_11_NO_3_	HMDB0000158	C00082	Up
M8	1.86	L-Leucine	132.102	[M+H]^+^	C_6_H_13_NO_2_	HMDB0000687	C00123	Down
M9	3.52	L-Tryptophan	203.0818	M-H	C_11_H_12_N_2_O_2_	HMDB0000929	C00078	Down
M10	3.96	Indole	118.0652	[M+H]^+^	C_8_H_7_N	HMDB0000738	C00463	Up
M11	4.03	Phenylacetylglutamine	263.1036	M-H	C_13_H_16_N_2_O_4_	HMDB0006344	C04148	Down
M12	4.22	Protoporphyrinogen IX	569.3142	[M+H]^+^	C_34_H_40_N_4_O_4_	HMDB0001097	C01079	Up
M13	4.29	Coproporphyrin III	653.2657	M-H	C_36_H_38_N_4_O_8_	HMDB0000570	C05770	Up
M14	4.38	Phenylalanylphenylalanine	313.1546	[M+H]^+^	C_18_H_20_N_2_O_3_	HMDB0013302		Up
M15	4.52	PE(18:4(6Z,9Z,12Z,15Z)/22:6(4Z,7Z,10Z,13Z,16Z,19Z))	784.4883	[M+H]^+^	C_45_H_70_NO_8_P	HMDB0009210	—	Up
M16	4.58	3,4-Dihydroxymandelic acid	183.0288	M-H	C_8_H_8_O_5_	HMDB0001866	C05580	Up
M17	4.8	Indoleacrylic acid	188.0707	M+H	C_11_H_9_NO_2_	HMDB0000734	—	Up
M18	5.08	Corticosterone	347.2215	M+H	C_21_H_30_O_4_	HMDB0001547	C02140	Up
M19	5.56	Taurocholate	516.2989	M+H	C_26_H_45_NO_7_S	HMDB0000036	C05122	Down
M20	5.64	Galactosylglycerol	253.093	M-H	C_9_H_18_O_8_	HMDB0006790	C05401	Down
M21	5.96	Bilirubin	585.2705	M+H	C_33_H_36_N_4_O_6_	HMDB0000054	C00486	Down
M22	6.08	Glycocholic acid	466.3163	M+H	C_26_H_43_NO_6_	HMDB0000138	C01921	Down
M23	6.44	Taurochenodeoxycholic acid	498.289	M-H	C_26_H_45_NO_6_S	HMDB0000951	C05465	Down
M24	6.66	LysoPA(0:0/18:2(9Z,12Z))	433.2356	M-H	C_21_H_39_O_7_P	HMDB0007852	—	Down
M25	6.83	Dodecanoylcarnitine	344.2794	M+H	C_19_H_37_NO_4_	HMDB0002250	—	Up
M26	6.88	Cholic acid	407.2796	M-H	C_24_H_40_O_5_	HMDB0000619	C00695	Up
M27	6.89	Biliverdin	583.2547	M+H	C_33_H_34_N_4_O_6_	HMDB0001008	C00500	Up
M28	7.15	Deoxycholylglycine	450.3213	M+H	C_26_H_43_NO_5_	HMDB0242414	—	Down
M29	7.28	N-Succinyl-L,L-2,6-diaminopimelate	289.1054	M-H	C_11_H_18_N_2_O_7_	HMDB0012267	C04421	Down
M30	7.28	13-Oxo-9,11-tridecadienoic acid	223.1333	M-H	C_13_H_20_O_3_	HMDB0034564	—	Down
M31	7.45	Deoxycholic acid	391.2849	M-H	C_24_H_40_O_4_	HMDB0000626	C04483	Down
M32	8.06	LysoPC(20:5(5Z,8Z,11Z,14Z,17Z)/0:0)	542.3217	M+H	C_28_H_48_NO_7_P	HMDB0010397	C04230	Down
M33	8.1	PE(22:4(7Z,10Z,13Z,16Z)/22:5(4Z,7Z,10Z,13Z,16Z))	840.5488	M-H	C_49_H_80_NO_8_P	HMDB0009603	C00350	Down
M34	8.12	Tetracosahexaenoic acid	357.2787	M+H	C_24_H_36_O_2_	HMDB0002007	—	Down
M35	8.24	Palmitoylcarnitine	400.342	M+H	C_23_H_45_NO_4_	HMDB0000222	C02990	Up
M36	8.32	4-Hydroxyphenylpyruvic acid	181.0497	M+H	C_9_H_8_O_4_	HMDB0000707	C01179	Down
M37	8.36	LysoPC(P-18:1(9Z)/0:0)	506.3601	M+H	C_26_H_52_NO_6_P	HMDB0010408	C04230	Down
M38	8.62	LysoPC(18:3(6Z,9Z,12Z)/0:0)	518.3218	M+H	C_26_H_48_NO_7_P	HMDB0010387	C04230	Down
M39	8.78	Prostaglandin E2	353.2298	M+H	C_20_H_32_O_5_	HMDB0001220	C00584	Up
M40	9.28	Androsterone glucuronide	467.2619	M+H	C_25_H_38_O_8_	HMDB0002829	C11135	Down
M41	10.1	LysoPC(P-18:0/0:0)	508.3763	M+H	C_26_H_54_NO_6_P	HMDB0013122	C04230	Down
M42	10.38	DG(16:0/LTE4/0:0)	752.5199	M+H	C_42_H_73_NO_8_S	HMDB0295467	—	Down
M43	10.53	16-Hydroxyhexadecanoic acid	271.2277	M-H	C_16_H_32_O_3_	HMDB0006294	C18218	Down
M44	10.66	PE(PGF1alpha/18:0)	820.5769	M+H	C_43_H_82_NO_11_P	HMDB0261187	—	Down
M45	10.67	17-beta-Estradiol-3-glucuronide	447.1998	M-H	C_24_H_32_O_8_	HMDB0006224	C05503	Up
M46	10.74	(9S,10S)-10-hydroxy-9-(phosphonooxy)octadecanoate	395.2199	M-H	C_18_H_37_O_7_P	HMDB0059632	C15989	Down
M47	10.75	Docosahexaenoic acid	327.2328	M-H	C_22_H_32_O_2_	HMDB0002183	C06429	Down
M48	10.97	Arachidonic acid	303.2327	M-H	C_20_H_32_O_2_	HMDB0001043	C00219	Down
M49	11.17	Linoleic acid	279.2326	M-H	C_18_H_32_O_2_	HMDB0000673	C01595	Down
M50	11.21	PC(14:0/18:2(9Z,12Z))	730.5375	M+H	C_40_H_76_NO_8_P	HMDB0007874	C00157	Up
M51	11.43	Docosapentaenoic acid (22n-3)	329.2483	M-H	C_22_H_34_O_2_	HMDB0006528	C16513	Down
M52	12.12	PC(22:6(4Z,7Z,10Z,13Z,16Z,19Z)/20:3(5Z,8Z,11Z))	856.5799	M+H	C_50_H_82_NO_8_P	HMDB0008737	C00157	Down
M53	12.14	PC(18:2(9Z,12Z)/22:6(4Z,7Z,10Z,13Z,16Z,19Z))	830.5686	M+H	C_48_H_80_NO_8_P	HMDB0008156	C00157	Down
M54	12.6	PE(20:4(5Z,8Z,11Z,14Z)/P-18:1(11Z))	748.5267	M-H	C_43_H_76_NO_7_P	HMDB0009413	C00350	Up
M55	14.65	PC(18:0/20:4(8Z,11Z,14Z,17Z))	810.5997	M+H	C_46_H_84_NO_8_P	HMDB0008049	C00157	Down
M56	14.72	PC(18:0/18:2(9Z,12Z))	786.5998	M+H	C_44_H_84_NO_8_P	HMDB0008039	C00157	Down

### 3.3 ATP1A1, SLC2A1, EEF2, and other 191 substances were identified as differential proteins of DHG

**Figure 3:**
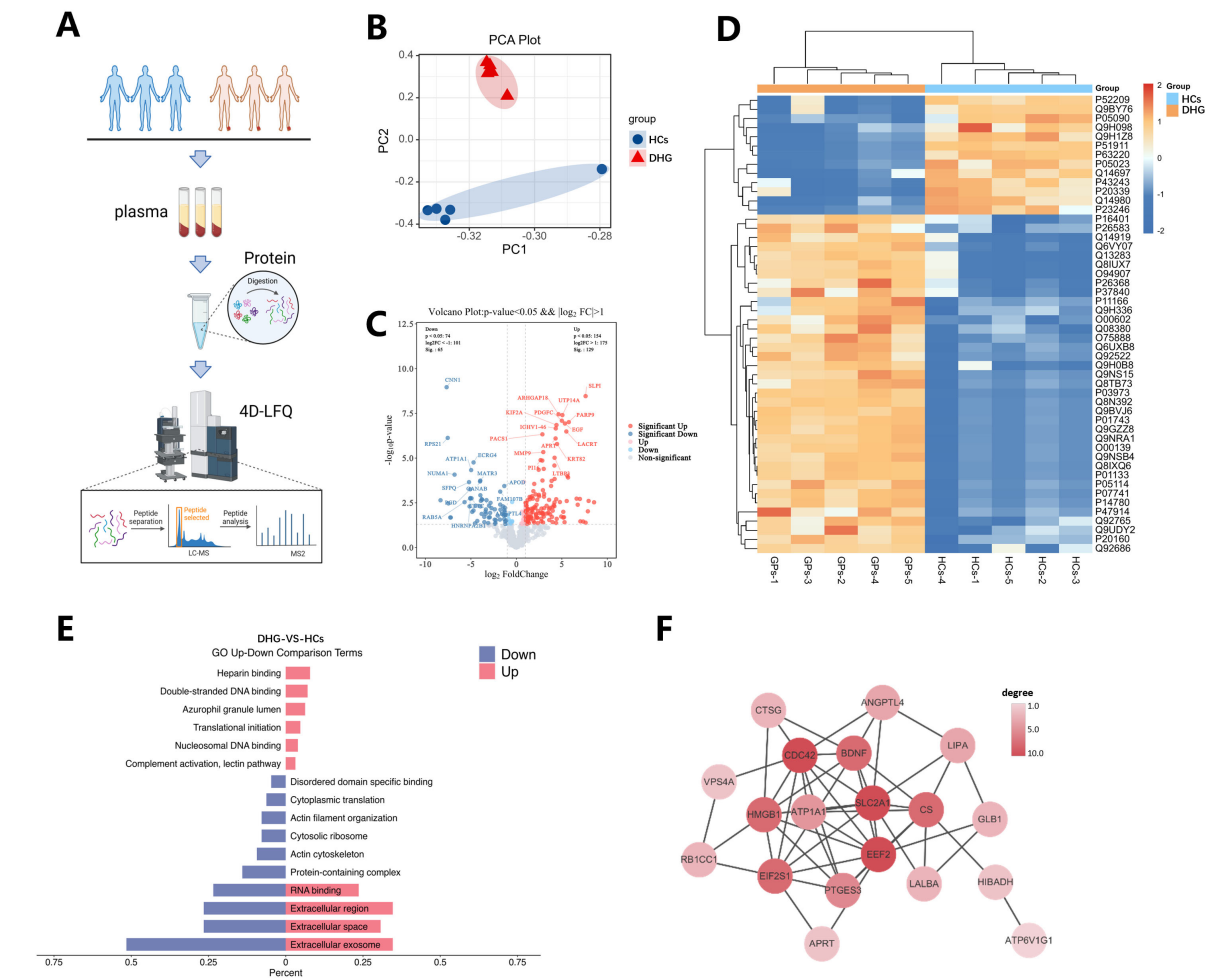
Screening and enrichment analysis of differential protein. **(A)** Flow chart of proteomics data acquisition; **(B)** PCA scatter plot showed that the protein data of healthy controls and DHG patients had good separation characteristics. **(C)** Volcano map of differential protein screening. **(D)** Heatmap of top 50 differential proteins (P value). **(E)** Comparison chart of up-down adjustment (The top 10 items with ListHits greater than 1 and the smallest up-down p-value of GO respectively). **(F)** The PPI network diagram of the top 25 proteins with connectivity.

For an unbiased understanding of the underlying molecular determinants that modulate the onset and progression of DHG in circulation, we randomly selected 50 serum samples in each group. Sets of 10 serum samples were then mixed into 1 loading sample, resulting in 5 loading samples per group (**Figure 3A**). In total, 1066 proteins were quantified across all 10 loading samples, and protein expression profiles were analyzed by PCA to compare differential expression between the DHG and HCs group. (**Figure 3B**). The proteins were screened based on the criteria of P < 0.05 and FC > 2.0/FC < 1/2.0 (**Figure 3C**); 194 differential proteins were obtained, and the heat map of the protein content of the top 50 proteins (in ascending order of P-value) is shown in **Figure 3D**. Differential proteins were analyzed for GO enrichment, and pathways in the three categories (biological process, cellular component, molecular function) were sorted by P-value. Select 10 items with ListHits greater than 1 and the smallest up-down p-value of GO respectively, and draw a comparison chart of up-down adjustment (**Figure 3E**). Homo sapiens was selected in the STRING database to analyze the differential proteins, and the PPI networks of the differential proteins was obtained. The top 25 proteins with connectivity were selected, and the connectivity of these proteins was recalculated and the network diagram was drawn (**Figure 3F**). Finally, nine core functional proteins with degree>5 were screened out: SLC2A1, EEF2, CDC42, CS, EIF2S1, HMGB1, BDNF, PTGES3 and ATP1A1 (**Supplementary Table 3**).

### 3.4 Bile secretion, protein digestion and absorption, mineral absorption and other 18 common pathways are associated with DHG symptoms by joint analysis

**Figure 4:**
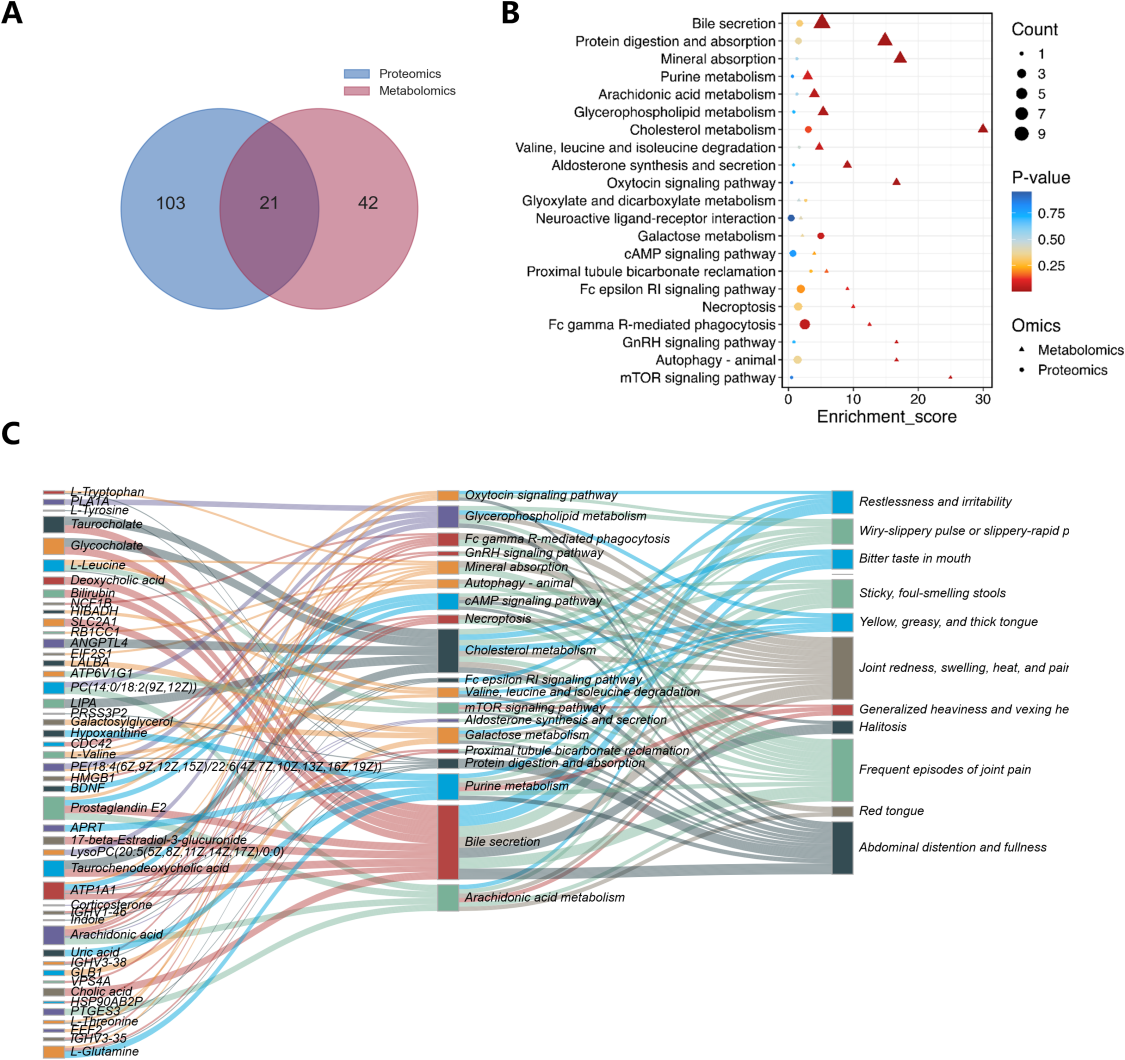
Analysis of metabolomics and proteomics common pathways and their association with DHG symptoms. **(A)** Venn plot of 21 pathways common to metabolomics and proteomics. **(B)** Bubble plot of the 21 common pathways. **(C)** Protein/metabolite-KEGG pathway-symptom association map screened out 18 common pathways (See **Supplementary table 5** for references).

In order to screen metabolites and protein which play a key role in DHG, and focus on its core biological pathway, we conducted a joint analysis of metabolomics and proteomics. At the expression level, we ranked the differential metabolites and differential proteins according to the P-value and selected the top 20 metabolites and proteins for correlation analysis (**Supplementary Figure 1**). The results showed that there was a high correlation between differential metabolites and differential proteins. At the pathway level, 63 pathways were enriched by metabolomics, 124 pathways were enriched by proteomics, and 21 pathways were common to both (**Figure 4A**). We screened for common pathways enriched by metabolomics and proteomics (**Figure 4B**) among them.

Dampness-heat syndrome is the most prevalent traditional TCM pattern observed clinically in gout. DHG presents with an acute onset, characterized by redness, swelling, heat, and pain in the joints as its primary manifestations, accompanied by systemic symptoms such as fever and heaviness in the body, bitter mouth, sticky stool and so on. To distinguish DHG from other TCM patterns of gout and precisely identify associated metabolites and proteins, we analyzed the correlation between the biological significance of the aforementioned 21 pathways and the 11 characteristic symptoms of DHG. Ultimately, this analysis yielded a differential protein/metabolite-pathway-symptom Sankey diagram. The results revealed that 18 pathways associated with DHG symptoms and could be traced upstream to the metabolites/proteins causing changes in the pathways, which revealed the molecular mechanism underlying DHG. (**Figure 4C**).

### 3.5 Focusing on the top4 metabolic pathway which is highly related to the main symptoms of DHG, 13 differential metabolites and 6 differential protein were screened out

**Figure 5:**
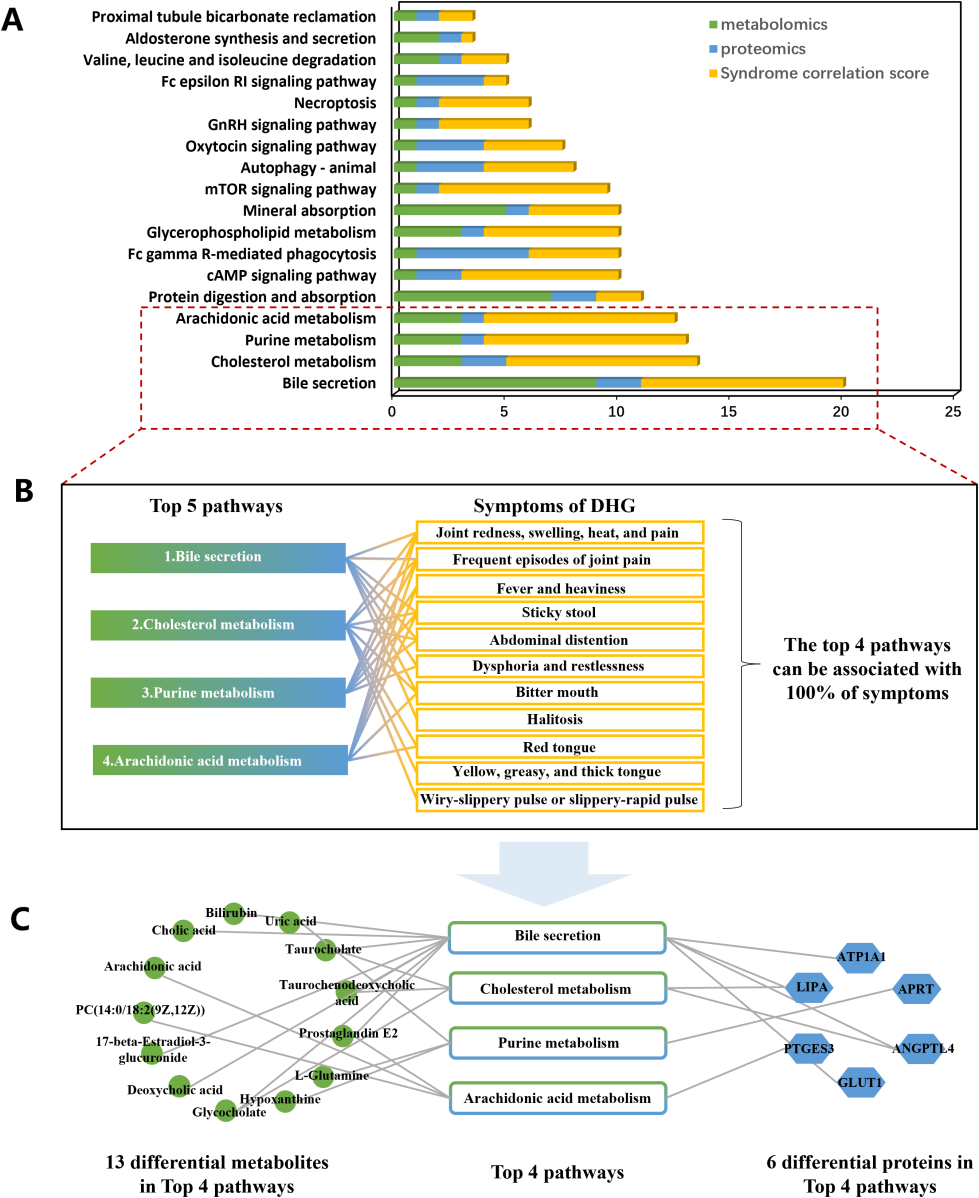
Screening of crucial pathways associated with DHG main symptoms. **(A)** Ranking map of enrichment scores of 18 common pathways got four crucial pathways. **(B)** Association map of the top four common pathways with main symptoms. **(C)** Differential metabolites and differential proteins associated with the top four common pathways.

To identify crucial pathways representative of DHG’s common symptoms, we integrated the results of pathway enrichment analysis with diagnostic symptom relevance scores to rank 18 pathways associated with DHG diagnostic symptoms (**Figure 5A**). According to diagnostic criteria, the diagnostic symptoms of DHG are categorized into primary symptoms, secondary symptoms, and tongue-pulse patterns (**Table 3**). Among these, having two main symptoms, or one main symptom and two secondary symptoms, combined with tongue and pulse diagnosis, can diagnose DHG. We assigned 3 points to main symptoms, 2 points to secondary symptoms, and 1 point to tongue and pulse diagnosis (**Supplementary Tables 4**, **5**). The results showed that the top 4 pathways could cover all 11 DHG diagnostic symptoms, which are Bile secretion, Cholesterol metabolism, Purine metabolism, Arachidonic acid metabolism, and Protein digestion and absorption (**Figure 5B**). Then, the 13 differential metabolites and 6 differential proteins in the top five pathways were extracted (**Figure 5C**). These metabolites were for biomarker screening and construct the diagnostic model, and these proteins were used for key target screening.

**Table 3:** Reference table for diagnosis of DHG.

Category	Symptoms
Primary symptoms	Joint redness, swelling, heat, and pain; frequent episodes of joint pain; fever and heaviness
Secondary symptoms	Dysphoria and restlessness; abdominal distension; bitter mouth; halitosis; sticky stool
Tongue-pulse patterns	Red tongue; yellow and greasy tongue coating; wiry-slippery pulse or slippery-rapid pulse

Diagnostic principle of DHG is to meet two main symptoms, or one main symptom and two secondary symptoms, combined with tongue and pulse diagnosis.

### 3.6 Deoxycholic acid, prostaglandin E2, bilirubin, uric acid, and hypoxanthine, identified by machine learning analysis as highly diagnostic biomarkers, served as the foundation for the successful DHG diagnostic model

**Figure 6:**
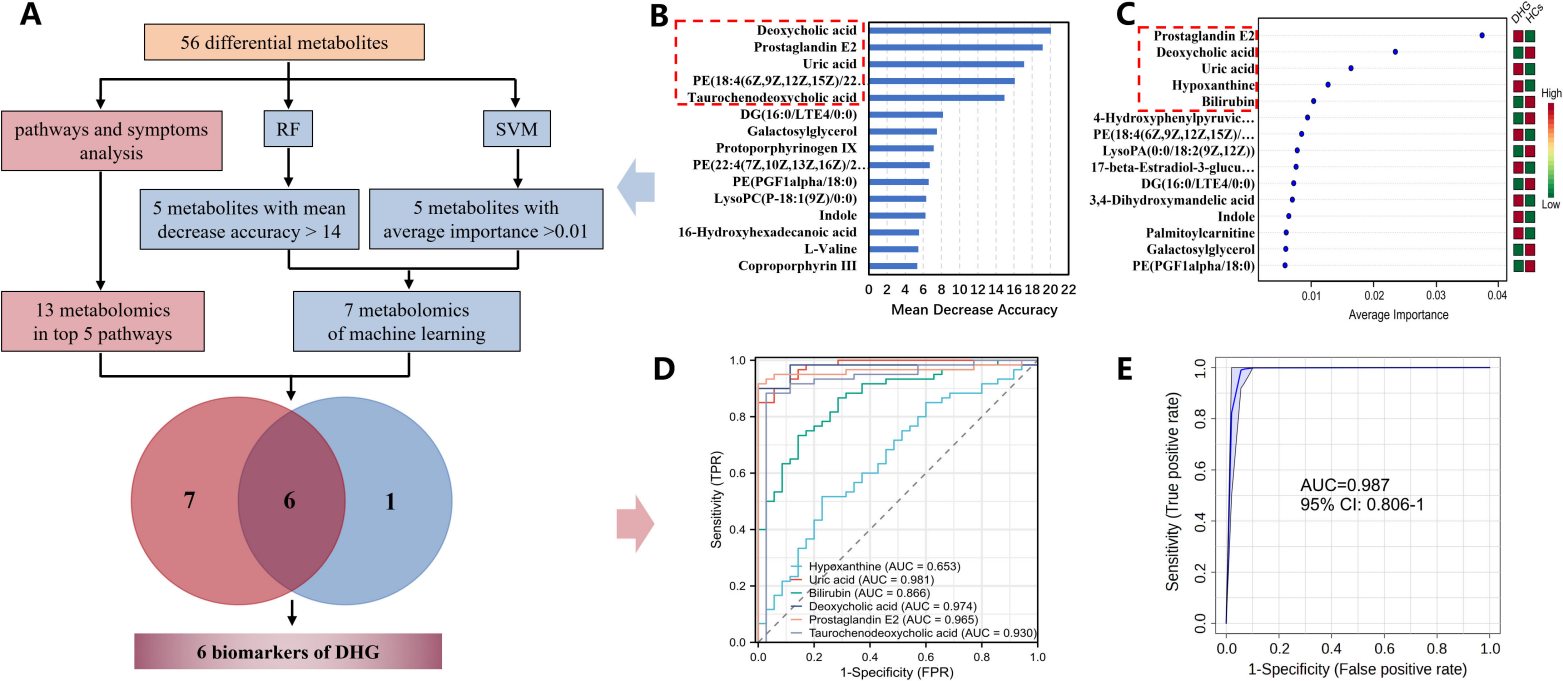
Screening of biomarkers. **(A)** flow chart of biomarker screening; **(B)** quantitative selection of effective features of random forest (screening the top five metabolites with mean decrease accuracy > 14); **(C)** quantitative selection of effective features of support vector machine (screening the top five metabolites with average importance >0.01); **(D)** individual ROC curves for six biomarkers in the validation cohort; **(E)** ROC curve based on the biomarker model created under the RF algorithm in the validation cohort.

To further refine and select the most predictive and stable biomarkers, we employed the RF algorithm performing 500 decision trees. Based on the results of Mean decrease accuracy, we ultimately identified 5 key metabolites (Deoxycholic acid, Prostaglandin E2, Uric acid, PE(18:4(6Z,9Z,12Z,15Z)/22:6(4Z,7Z,10Z,13Z,16Z,19Z)), 4-Hydroxyphenylpyruvic acid) with mean decrease accuracy > 14 (**Figure 6B**). The SVM feature importance analysis revealed 15 key metabolites that contributed significantly to the distinction between DHG and HCs groups (**Figure 6C**). The top important features were prostaglandin E2, deoxycholic acid, uric acid, hypoxanthine, and bilirubin (Average importance >0.01). Among these, prostaglandin E2 showed the highest importance (average importance ≈0.04) and was highly expressed in the HCs group but low in DHG. Eventually, we screened out five differential metabolites with high correlation with the biological mechanism of DHG symptoms and high diagnostic ability as biomarkers of DHG: hypoxanine, uric acid, bilirubin, deoxycholic acid, and prostaglandin E2 (**Figure 6A**).

**Figure 7:**
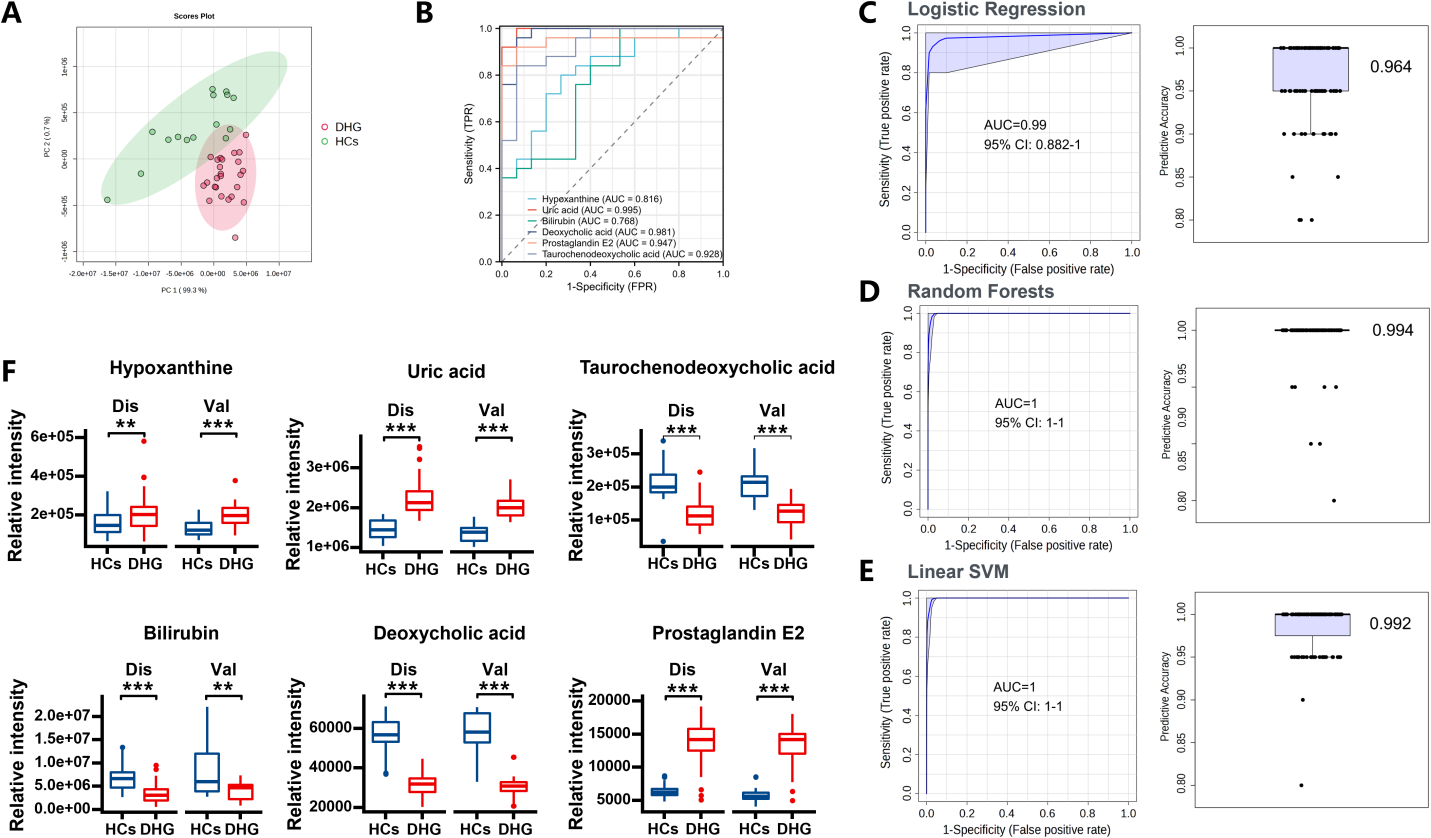
Verification of DHG diagnostic model. **(A)** PCA scatter plot based on 6 biomarkers in validation cohort; **(B)** individual ROC curves for six biomarkers in the validation cohort; **(C)** ROC curve of the biomarker model created under the LR algorithm and prediction class probability diagram of all samples in the validation cohort. **(D)** ROC curve based on the biomarker model created under the RF algorithm and the prediction class probability graph of all samples in the validation cohort. **(E)** ROC curve of the biomarker model created under the SVM algorithm and prediction class probability graph of all samples in the validation cohort. **(F)** the box diagrams of six biomarkers’ expression in analysis set and verification set (**P<0.01, *** P<0.001).

In order to evaluate the diagnostic ability of biomarkers for DHG, we used the pROC package to analyze the expression data of six biomarkers respectively to reflect their sensitivity and specificity. The results show that prostaglandin E2, deoxycholic acid, Taurochenodeoxycholic acid and uric acid have strong diagnostic abilities (AUC>9.0), while hypoxanthine and bilirubin have normal diagnostic abilities (0.65<AUC<0.87) (**Figure 6D**). The diagnosis of DHG needs to aggregate multiple symptoms, and the biomarkers of a single indicator are not sufficient to represent the overall changes in DHG. Therefore, we analyzed the ROC of six biomarkers as a whole, and its AUC value reached 0.987 (**Figure 6E**). Then we verify the diagnostic ability of six biomarker in the validation cohorts. PCA analysis showed that the differential expression of six biomarkers could be successfully separated HCs and DHG groups (**Figure 7A**). The ROC curves in validation cohorts of the above biomarkers were calculated, and the AUCs were 0.768 to 0.995 (**Figure 7B**). To verify the diagnostic value of indicator combination, 6 biomarkers were subjected to diverse multivariate analyses using LR, RF, and SVM algorithms within the machine learning model. All three algorithm models exhibited the AUC > 0.990 (**Figures 7C-E**). The expression of biomarker in set A and set B is stable and the trend is the same (**Figure 7F**). Consequently, these 6 biomarkers have the excellent predictive and stable, effectively distinguishing between HCs and DHG patients.

### 3.7 LIPA, GLUT1, ATP1A1, APRT, ANGPTL4 and PTGES3 are associated with the main symptoms and identified as potential therapeutic targets of DHG

Six proteins were differentially expressed in four crucial pathways related to DHG: lysosomal acid lipase A (LIPA), glucose transporters 1 (GLUT1), ATPase Na+/K+ transporting subunit alpha 1 (ATP1A1), adenine phosphoribosyltransferase (APRT), angiopoietin-related protein 4 (ANGPTL4), and Prostaglandin E synthase 3 (PTGES3). The expression trend of six differential proteins was analyzed, and it was found that 3 proteins were increased and 3 proteins were decreased in DHG patients.

## 4 Discussion

**Figure 8:**
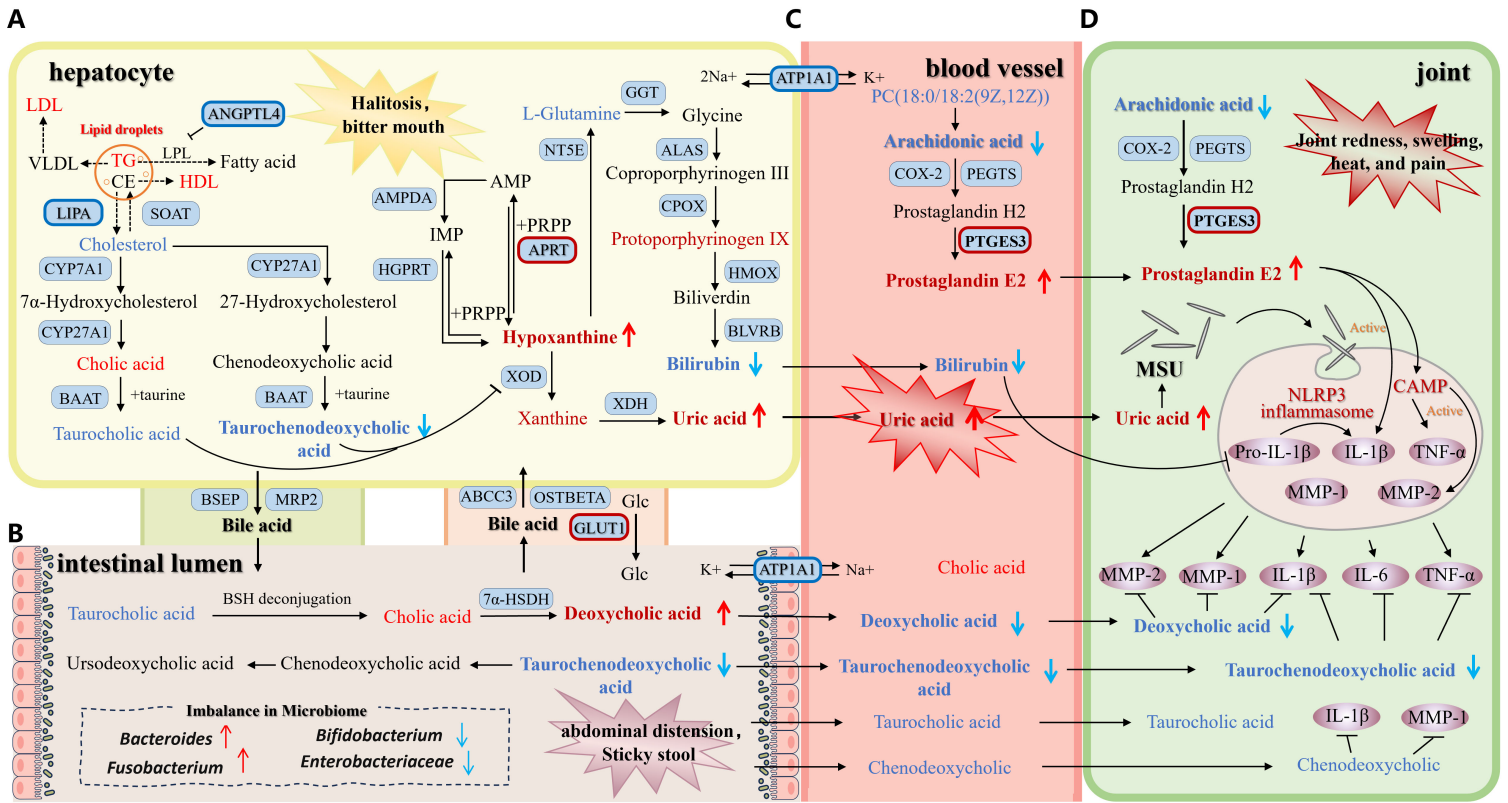
Schematic diagram of pathological mechanism of DHG based on biomarkers and crucial pathways. The metabolite of bold font is biomarker. The red font indicates a significant upward trend in DHG patients, and the blue font indicates a significant downward trend in DHG patients. Blue squares represent proteins. Proteins in bold are key potential therapeutic targets for DHG. The red outline indicates increased protein expression, while the blue outline indicates decreased protein expression. **(A)** The processes in the hepatocyte, including bile secretion, cholesterol metabolism and purine metabolism pathway. **(B)** The processes in the intestinal lumen, including bile secretion pathway and its effect on the microbiome. **(C)** The processes in the blood vessel, including bile acid transport and arachidonic acid metabolic pathway. **(D)** The processes in the joint, including arachidonic acid metabolic pathway and inflammatory reaction process of gout.

Current methods for diagnosing gout rely on elevated serum uric acid levels combined with typical clinical manifestations and the identification of urate crystals in synovial fluid (23). However, serum uric acid levels may be normal during an arthritis flare. Moreover, the high cost of magnetic resonance imaging and the discomfort associated with synovial fluid aspiration limit the clinical utility of urate crystal identification (24). Critically, neither serum uric acid levels nor synovial urate crystals reliably enable early gout diagnosis or predict the timing of acute inflammatory attacks. The diagnosis of DHG requires integrating syndrome manifestations based on the confirmation of gout, making the process more complex and lacking objective and quantifiable indicators (12). Therefore, we selected DHG patients from Guangzhou, a high-incidence area for this condition, and strictly placed healthy individuals in the healthy control group. We conducted metabolomic and proteomic analyses on blood samples from both groups to identify and explain the pathological mechanism of DHG from the perspective of protein expression and metabolic characteristics. A biomarker that can be used to diagnose DHG as an objective diagnostic index should be identified. Moreover, potential therapeutic targets of DHG should be identified, and an accurate diagnostic and treatment model of DHG should be constructed. In this study, 56 differentially abundant metabolites and 239 differentially abundant proteins were found between DHG patients and healthy controls, and 21 common pathways were identified through enrichment analysis. To further investigate the crucial pathways associated with DHG, we screened four core pathways representing DHG based on the enrichment scores of the pathways and the degree of correlation with the clinical symptoms of DHG. Using omics data integration and machine learning pattern recognition methods, we identified six biomarkers that can distinguish patients with DHG from healthy people. Six biomarkers are associated with 11 symptoms and signs of DHG through four crucial pathways. Therefore, the joint diagnosis of these six biomarkers can be used to comprehensively monitor DHG. Additionally, six differential proteins in four crucial pathways, which constitute a potential target group to reverse metabolic abnormalities and improve DHG syndrome, were also identified. Based on the acquired omics data, we systematically elucidated the pathogenesis of DHG and potential therapeutic targets (**Figure 8**).

Purine metabolism is the pathway underlying uric acid production, and the imbalance of this metabolic pathway interacts through many links to drive the deposition of urate crystals and inflammation. In this study, hypoxanthine levels increased, L-glutamine levels decreased, and uric acid accumulated in DHG patients. The abnormal accumulation of hypoxanthine provides the direct precursor of uric acid overproduction. When the content of hypoxanthine in the body increases, it is converted into xanthine and eventually produces uric acid under the catalysis of xanthine oxidase (XOD) (25). The continuous supersaturation of uric acid triggers a cascade of reactions involving crystal deposition and inflammation. When the blood uric acid concentration is greater than 480 μmol/L (about 8 mg/dL), monosodium urate (MSU) precipitates in low-temperature/acidic microenvironments, such as synovial fluid (26). After MSU crystals are engulfed by macrophages, they trigger the explosive release of interleukin-1β (IL-1β), tumor necrosis factor-α (TNF-α), and MMPs (such as MMP-1 and MMP-2). by activating NOD-like receptor thermal protein domain-associated protein 3 (NLRP3) inflammatory corpuscles (27). This process leads to substantial infiltration of neutrophils, vasodilation, tissue edema, and hyperalgesia, which results in local redness, swelling, heat, pain, and systemic inflammatory reactions in DHG patients, and MMPs mediate the degradation of joint tissue (28). Elevated serum uric acid levels also trigger a systemic inflammatory response (29). Inflammatory factors (IL-1β, TNF-α, and IL-6) may cross the blood-brain barrier or activate vagal afferent pathways, impacting brain regions involved in mood regulation (such as the prefrontal cortex and amygdala) (30). These cytokines disrupt the metabolism and balance of neurotransmitters like serotonin and dopamine, leading to impaired emotional regulation. This manifests in DHG patients as symptoms such as restlessness and anxiety. Furthermore, inflammatory factors like TNF-α can compromise the integrity of tight junctions between intestinal epithelial cells, increasing gut permeability (31, 32). This heightened permeability may facilitate the translocation of endotoxins (e.g., lipopolysaccharide) into the bloodstream, exacerbating systemic inflammation and establishing a vicious cycle (33, 34). Gut dysbiosis frequently accompanies this process and directly contributes to symptoms like abdominal bloating, excessive gas production, and abnormal stool consistency. Our findings indicate significantly upregulated APRT expression in DHG patients. APRT overexpression disrupts purine metabolism homeostasis through multiple mechanisms. Its hyperactivity rapidly depletes cellular PRPP reserves by driving excessive adenine salvage (35). This PRPP scarcity critically impairs HGPRT function, preventing the recycling of hypoxanthine into IMP (36, 37). Concurrently, the surplus AMP generated by APRT undergoes stepwise catabolism: AMP is deaminated to IMP, hydrolyzed to inosine, and finally cleaved to hypoxanthine. Consequently, hypoxanthine production escalates while its primary salvage pathway remains blocked (38). The resulting intracellular hypoxanthine accumulation drives metabolic flux toward oxidation. Xanthine oxidase sequentially converts the accumulated hypoxanthine first into xanthine and subsequently into uric acid, culminating in pathological uric acid elevation. Consequently, reducing this APRT overexpression may help decrease hypoxanthine and uric acid production, thereby alleviating frequent joint inflammation and improving symptoms such as abdominal distention, sticky stool, dysphoria and restlessness. This positions APRT as a promising target for DHG treatment (**Figures 8A, B**).

We observed that patients with DHG exhibit significant abnormalities in bile acid metabolism, primarily involving the key pathways of bile secretion and cholesterol metabolism. Bile acids (BAs) are among the main components of bile, and they are generally referred to as cholanic acids. They are synthesized from cholesterol through a series of enzymatic reactions in the liver. Cholesterol is mediated by cholesterol 7α-hydroxylase (CYP7A1) and sterol 27-hydroxylase (CYP27A1) in liver parenchymal cells to generate primary BAs (including cholic acid, chenodeoxycholic acid, etc.), which are secreted into the intestine and converted into secondary BAs (deoxycholic acid and lithocholic acid) under the action of intestinal microorganisms. The main symptoms of DHG include high uric acid levels, frequent episodes of joint pain, sticky and foul-smelling stools, and abdominal distention (12, 39). It leads to poor lipid digestion and absorption and an imbalance of the intestinal flora, which leads to sticky and foul-smelling stools (40). BAs can reduce the expression of xanthine oxidase (XOD) and reduce the production of uric acid. However, in hyperuricemia, farnesoid X receptor (FXR) in the liver is abnormally activated, which leads to the inhibition of CYP7A1 expression, thereby reducing the conversion rate of cholesterol into BAs and continuously increasing uric acid levels (41, 42). Additionally, chenodeoxycholic acid can inhibit the release of inflammatory factors such as MMP-1, IL-1β, and PGE2 in synovial fluid (43), and taurochenodeoxycholic acid can reduce apoptosis and improve osteoarthritis by decreasing TNF-α, IL-1β, and IL-6 levels through the activity of nuclear factor kappa light chain enhancer of activated B cells(NF-κB) (44). Deoxycholic acid markedly reduces the release of MMP-1, MMP-3, and IL-1β in serum to inhibit inflammatory responses (45). However, in DHG patients, BAs such as taurochenodeoxycholic acid and deoxycholic acid are significantly reduced, which may be an important reason for the recurrence of joint inflammation. The decrease in the blood deoxycholic acid concentration in DHG patients may be related to the obstruction of its transport. A high concentration of deoxycholic acid in the intestine can aggravate the imbalance of the intestinal flora (such as a decrease in the relative abundance of *Bifidobacterium* and Enterobacteriaceae and an increase in the relative abundance of *Fusobacterium* and *Bacteroides*), which leads to abdominal distension and sticky stool in DHG patients (39, 46, 47). ANGPTL4 is a crucial regulator of lipid metabolism, functioning as a “lipid gatekeeper” that controls the distribution and utilization of fats. Its primary mechanism involves the potent inhibition of lipoprotein lipase (LPL), the enzyme responsible for hydrolyzing triglycerides in circulating lipoproteins (48, 49). LIPA is a key lysosomal enzyme that hydrolyzes intracellular cholesteryl esters into free cholesterol (50). This free cholesterol is subsequently transported to the endoplasmic reticulum, where it serves as a substrate for cholesterol 7α-hydroxylase (CYP7A1), which catalyzes the formation of 7α-hydroxycholesterol which is the rate-limiting step in bile acid synthesis (51, 52). Deficiencies in ANGPTL4 and LIPA contribute to systemic lipid accumulation and reduce the availability of free cholesterol for bile acid synthesis, leading to disrupted bile acid metabolism. Therefore, modulating these proteins may help mitigate abnormal elevations in TC and TG in DHG patients. Enhancing bile acid biosynthesis through this modulation could increase the production of specific bile acids such as taurochenodeoxycholic acid and deoxycholic acid, thereby DHG symptoms including recurrent joint pain, sticky and foul-smelling stools, and abdominal distention. Additionally, as a transmembrane ATPase, ATP1A1 hydrolyzes ATP to maintain sodium-potassium gradients and power the active transport of bile acids, lipids, and amino acids (53). GLUT1, the ubiquitously expressed glucose transporter, helps maintain blood glucose stability and energy supply, and may supports bile acid reabsorption (54). Targeting ATP1A1 and GLUT1 may therefore remodel bile acid metabolism by enhancing both active transport and reabsorption processes, offering a potential therapeutic strategy for relieving DHG-related symptoms. Furthermore, correcting underlying impairments in energy metabolism could provide additional benefit for manifestations associated with dampness-heat syndrome (**Figures 8A, B, D**).

Arachidonic acid (AA), a polyunsaturated fatty acid predominantly esterified in membrane phospholipids, serves as the precursor for various proinflammatory eicosanoids. Upon enzymatic conversion by cyclooxygenase (COX) and lipoxygenase (LOX) pathways, AA yields potent inflammatory mediators including prostaglandins (e.g., PGE2) and leukotrienes (e.g., LTB4), which orchestrate inflammatory responses and immune activation (55, 56). In DHG patients, MSU crystals activate macrophages, inducing cytosolic phospholipase A_2_ (Cpla2) to release AA from membrane phospholipids. The liberated AA undergoes rapid conversion to PGE2 through the coordinated action of cyclooxygenase-2 (COX-2) and microsomal prostaglandin E synthase-1 (mPGES-1) (57, 58). PGE2 binds to EP2/EP4 receptors, activating adenylate cyclase (AC) and increasing intracellular cAMP levels. These effects mediate vasodilation, increased vascular permeability, and pain sensitization, exacerbating joint redness, swelling, heat, and pain (57, 59). In IFN-γ-activated and TNF-α-activated macrophages, PGE2 phosphorylates the transcription factor CREB through the cAMP/PKA pathway, amplifying inflammatory cytokines (e.g., IL-1β and IL-6) and forming a proinflammatory feedback loop (58, 60). MMP-mediated tissue damage releases endogenous danger molecules, activating the NLRP3 inflammasome and promoting the maturation of IL-1β. Together with PGE2 and TNF-α, this forms an “AA-PGE2-MMP-inflammatory cytokine” cascade storm, perpetuating acute inflammation and causing osteoarticular damage (57, 61). Central to this pathogenic cascade is PTGES3, the terminal enzyme converting prostaglandin H2 (PGH2) to PGE2 in the AA metabolic pathway (62). Its upregulated expression directly accelerates this inflammatory cascade, contributing to both chronic inflammation persistence and acute flare recurrence in DHG. These molecular events underlie the characteristic clinical manifestations of joint inflammation (redness, swelling, heat and pain) and systemic symptoms (altered bowel consistency, dysphoria, and restlessness) observed in DHG patients. The therapeutic benefit of PTGES3 regulation has been established in rheumatoid arthritis (63). Investigating its role in gouty arthritis and its capacity to improve damp-heat syndrome symptoms is a worthy research direction (**Figures 8C, D**).

This study focused on patients with DHG from the Guangzhou region, a geographic area characterized by its humid climate and dietary habits rich in high-purine foods (e.g., seafood, alcoholic beverages), which contribute to the high prevalence of DHG. The biomarkers identified in this context are therefore characteristic manifestations and clinically relevant for the DHG. Future studies will consider incorporating external validation cohorts from diverse geographical regions to evaluate the generalizability of these biomarkers across populations with varying climatic and dietary backgrounds. Additionally, future studies should compare other arthritic conditions (e.g., osteoarthritis and rheumatoid arthritis) and other TCM gout syndromes as controls to further confirm the specificity of biomarkers and targets for DHG. Such efforts would enhance the broader applicability of our findings and facilitate their translation into clinical practice across different regions. To further validate identified targets, we plan to establish DHG animal models and administer classical TCM formulations (e.g., Simiao Pill, Tongfengding) for whole-organism efficacy validation, concurrently characterizing core bioactive constituents. Subsequent cellular and animal models with target gene knockdown/overexpression will precisely evaluate candidate compounds. This integrated approach enables screening of targeted bioactive monomers or optimized polypharmacological combinations, which is critical step for developing mechanism-based TCM therapeutics and elucidating their mode of action. Ultimately, these investigations will support the advancement of precision diagnostics and targeted interventions for DHG.

## 5 Conclusions

The occurrence and development of DHG are associated with a multi-indicator diagnostic model reflecting inherent TCM characteristics. This model utilizes Hypoxanthine, Prostaglandin E2, Uric acid, Deoxycholic acid, Taurochenodeoxycholic acid, and Bilirubin as diagnostic indicators. These biomarkers are linked to four crucial metabolic pathways: Bile secretion, Cholesterol metabolism, Purine metabolism, and Arachidonic acid metabolism. Furthermore, the proteins ATP1A1, APRT, ANGPTL4, GLUT1, PTGES3, and LIPA represent potential therapeutic targets for DHG treatment.

## Data Availability

The datasets presented in this study can be found in online repositories. The names of the repository/repositories and accession number(s) can be found below: MTBLS12820 (Metabolights) and PXD067027 (ProteomeXchange).
