# Supplementary material for: Integrated multi-omics analysis of dampness-heat gout reveals diagnostic biomarkers and therapeutic targets
Source: Front Immunol. 2026 Feb 6;17:1677920. doi: 10.3389/fimmu.2026.1677920 (PMC12921442; doi:10.3389/fimmu.2026.1677920)
Supplement: Supplementary file 1 [file Table1.docx]

Supplementary table 1. the elution gradient of LC-MS/MS high resolution mass spectrometry

| Time（min） | Gradient |
| --- | --- |
| 0 | 2% B |
| 45 | 22% B |
| 50 | 37% B |
| 55 | 80% B |
| 60 | 80% B |

Note: phase A (0.1% formic acid in water) and phase B (0.1% formic acid in acetonitrile)

Supplementary Table 2. KEGG enrichment analysis results of differential metabolites

| **id** | **Term** | **ListHits** | **ListTotal** | **PopHits** | **p-value** | **Enrichment_score** |
| --- | --- | --- | --- | --- | --- | --- |
| hsa04974 | Protein digestion and absorption | 7 | 35 | 47 | 2.52274E-07 | 14.88510638 |
| hsa04978 | Mineral absorption | 5 | 35 | 29 | 7.45536E-06 | 17.23152709 |
| hsa04976 | Bile secretion | 9 | 35 | 174 | 3.48965E-05 | 5.169458128 |
| hsa04979 | Cholesterol metabolism | 3 | 35 | 10 | 0.000105008 | 29.98285714 |
| hsa04923 | Regulation of lipolysis in adipocytes | 3 | 35 | 14 | 0.000309882 | 21.41632653 |
| hsa00400 | Phenylalanine, tyrosine and tryptophan biosynthesis | 4 | 35 | 34 | 0.000314958 | 11.75798319 |
| hsa00120 | Primary bile acid biosynthesis | 4 | 35 | 47 | 0.001104481 | 8.505775076 |
| hsa00290 | Valine, leucine and isoleucine biosynthesis | 3 | 35 | 23 | 0.001417352 | 13.03602484 |
| hsa00591 | Linoleic acid metabolism | 3 | 35 | 28 | 0.002533501 | 10.70816327 |
| hsa01040 | Biosynthesis of unsaturated fatty acids | 4 | 35 | 74 | 0.005883479 | 5.402316602 |
| hsa04921 | Oxytocin signaling pathway | 2 | 35 | 12 | 0.006028808 | 16.65714286 |
| hsa04726 | Serotonergic synapse | 3 | 35 | 42 | 0.00806779 | 7.13877551 |
| hsa02010 | ABC transporters | 5 | 35 | 139 | 0.011442249 | 3.595066804 |
| hsa00860 | Porphyrin metabolism | 5 | 35 | 148 | 0.014743233 | 3.376447876 |
| hsa00564 | Glycerophospholipid metabolism | 3 | 35 | 56 | 0.017709418 | 5.354081633 |
| hsa04925 | Aldosterone synthesis and secretion | 2 | 35 | 22 | 0.019820679 | 9.085714286 |
| hsa04136 | Autophagy - other | 1 | 35 | 3 | 0.029726226 | 33.31428571 |
| hsa00590 | Arachidonic acid metabolism | 3 | 35 | 75 | 0.037928519 | 3.997714286 |
| hsa04150 | mTOR signaling pathway | 1 | 35 | 4 | 0.039442844 | 24.98571429 |
| hsa00350 | Tyrosine metabolism | 3 | 35 | 78 | 0.041874714 | 3.843956044 |
| hsa04750 | Inflammatory mediator regulation of TRP channels | 2 | 35 | 35 | 0.047091213 | 5.711020408 |
| hsa04140 | Autophagy - animal | 1 | 35 | 6 | 0.058593318 | 16.65714286 |
| hsa04912 | GnRH signaling pathway | 1 | 35 | 6 | 0.058593318 | 16.65714286 |
| hsa04916 | Melanogenesis | 1 | 35 | 6 | 0.058593318 | 16.65714286 |
| hsa00280 | Valine, leucine and isoleucine degradation | 2 | 35 | 42 | 0.06526133 | 4.759183673 |
| hsa00140 | Steroid hormone biosynthesis | 3 | 35 | 99 | 0.074964292 | 3.028571429 |
| hsa04666 | Fc gamma R-mediated phagocytosis | 1 | 35 | 8 | 0.07737269 | 12.49285714 |
| hsa04724 | Glutamatergic synapse | 1 | 35 | 8 | 0.07737269 | 12.49285714 |
| hsa00230 | Purine metabolism | 3 | 35 | 101 | 0.078588741 | 2.968599717 |
| hsa00260 | Glycine, serine and threonine metabolism | 2 | 35 | 48 | 0.08240284 | 4.164285714 |
| hsa00360 | Phenylalanine metabolism | 2 | 35 | 49 | 0.085384044 | 4.079300292 |
| hsa04727 | GABAergic synapse | 1 | 35 | 9 | 0.086625399 | 11.1047619 |
| hsa04730 | Long-term depression | 1 | 35 | 9 | 0.086625399 | 11.1047619 |
| hsa04217 | Necroptosis | 1 | 35 | 10 | 0.095787942 | 9.994285714 |
| hsa04664 | Fc epsilon RI signaling pathway | 1 | 35 | 11 | 0.10486117 | 9.085714286 |
| hsa04917 | Prolactin signaling pathway | 1 | 35 | 11 | 0.10486117 | 9.085714286 |
| hsa04625 | C-type lectin receptor signaling pathway | 1 | 35 | 12 | 0.113845931 | 8.328571429 |
| hsa04728 | Dopaminergic synapse | 1 | 35 | 12 | 0.113845931 | 8.328571429 |
| hsa04611 | Platelet activation | 1 | 35 | 14 | 0.131553388 | 7.13877551 |
| hsa00240 | Pyrimidine metabolism | 2 | 35 | 64 | 0.133574529 | 3.123214286 |
| hsa00470 | D-Amino acid metabolism | 2 | 35 | 67 | 0.143854895 | 2.98336887 |
| hsa04270 | Vascular smooth muscle contraction | 1 | 35 | 16 | 0.148916921 | 6.246428571 |
| hsa04924 | Renin secretion | 1 | 35 | 17 | 0.157471748 | 5.878991597 |
| hsa04964 | Proximal tubule bicarbonate reclamation | 1 | 35 | 17 | 0.157471748 | 5.878991597 |
| hsa00130 | Ubiquinone and other terpenoid-quinone biosynthesis | 2 | 35 | 71 | 0.157818921 | 2.815291751 |
| hsa00563 | Glycosylphosphatidylinositol (GPI)-anchor biosynthesis | 1 | 35 | 19 | 0.174331522 | 5.260150376 |
| hsa00910 | Nitrogen metabolism | 1 | 35 | 19 | 0.174331522 | 5.260150376 |
| hsa00380 | Tryptophan metabolism | 2 | 35 | 83 | 0.201079921 | 2.408261618 |
| hsa00220 | Arginine biosynthesis | 1 | 35 | 23 | 0.207073479 | 4.345341615 |
| hsa00430 | Taurine and hypotaurine metabolism | 1 | 35 | 24 | 0.215059789 | 4.164285714 |
| hsa04913 | Ovarian steroidogenesis | 1 | 35 | 24 | 0.215059789 | 4.164285714 |
| hsa04024 | cAMP signaling pathway | 1 | 35 | 25 | 0.222967938 | 3.997714286 |
| hsa00250 | Alanine, aspartate and glutamate metabolism | 1 | 35 | 28 | 0.246230804 | 3.569387755 |
| hsa00750 | Vitamin B6 metabolism | 1 | 35 | 29 | 0.253833663 | 3.446305419 |
| hsa00770 | Pantothenate and CoA biosynthesis | 1 | 35 | 30 | 0.261362006 | 3.331428571 |
| hsa04216 | Ferroptosis | 1 | 35 | 31 | 0.268816542 | 3.223963134 |
| hsa00730 | Thiamine metabolism | 1 | 35 | 31 | 0.268816542 | 3.223963134 |
| hsa00561 | Glycerolipid metabolism | 1 | 35 | 38 | 0.318989889 | 2.630075188 |
| hsa00592 | alpha-Linolenic acid metabolism | 1 | 35 | 44 | 0.359320008 | 2.271428571 |
| hsa00052 | Galactose metabolism | 1 | 35 | 46 | 0.372240333 | 2.172670807 |
| hsa00071 | Fatty acid degradation | 1 | 35 | 50 | 0.397326024 | 1.998857143 |
| hsa04080 | Neuroactive ligand-receptor interaction | 1 | 35 | 52 | 0.409500942 | 1.921978022 |
| hsa00630 | Glyoxylate and dicarboxylate metabolism | 1 | 35 | 62 | 0.466879514 | 1.611981567 |

Supplementary Table 3. Protein PPI interaction analysis results

| Accession | Gene Name | FoldChange | Degree | degree |
| --- | --- | --- | --- | --- |
| P11166 | SLC2A1 | 5.65493327 | 10 | 10 |
| P13639 | EEF2 | 4.24995628 | 10 | 10 |
| P60953 | CDC42 | 0.195493426 | 10 | 10 |
| O75390 | CS | 2.217650476 | 8 | 8 |
| P05198 | EIF2S1 | 5.130608705 | 8 | 8 |
| P09429 | HMGB1 | 2.602882163 | 8 | 8 |
| P23560 | BDNF | 2.31313906 | 8 | 8 |
| Q15185 | PTGES3 | 2.033143437 | 6 | 6 |
| P05023 | ATP1A1 | 0.031880057 | 5 | 5 |
| P38571 | LIPA | 0.098017693 | 4 | 4 |
| Q9BY76 | ANGPTL4 | 0.064465183 | 4 | 4 |
| P00709 | LALBA | 4.000987163 | 3 | 3 |
| P08311 | CTSG | 3.407538601 | 3 | 3 |
| P16278 | GLB1 | 0.097724764 | 3 | 3 |
| Q8TDY2 | RB1CC1 | 12.99877711 | 3 | 3 |
| P07741 | APRT | 19.83818316 | 2 | 2 |
| P31937 | HIBADH | 0.006499759 | 2 | 2 |
| Q9UN37 | VPS4A | 2.933803001 | 2 | 2 |
| O75348 | ATP6V1G1 | 0.139690905 | 1 | 1 |

Supplementary Table 4. Scoring table of 18 pathways related to DHG diagnosis symptoms

| Pathways | differential metabolites | differential protein | syndrome correlation score | total score |
| --- | --- | --- | --- | --- |
| Bile secretion | 9 | 2 | 9 | 20 |
| Cholesterol metabolism | 3 | 2 | 8.5 | 13.5 |
| Purine metabolism | 3 | 1 | 9 | 13 |
| Arachidonic acid metabolism | 3 | 1 | 8.5 | 12.5 |
| Protein digestion and absorption | 7 | 2 | 2 | 11 |
| cAMP signaling pathway | 1 | 2 | 7 | 10 |
| Fc gamma R-mediated phagocytosis | 1 | 5 | 4 | 10 |
| Glycerophospholipid metabolism | 3 | 1 | 6 | 10 |
| Mineral absorption | 5 | 1 | 4 | 10 |
| mTOR signaling pathway | 1 | 1 | 7.5 | 9.5 |
| Autophagy - animal | 1 | 3 | 4 | 8 |
| Oxytocin signaling pathway | 1 | 3 | 3.5 | 7.5 |
| GnRH signaling pathway | 1 | 1 | 4 | 6 |
| Necroptosis | 1 | 1 | 4 | 6 |
| Fc epsilon RI signaling pathway | 1 | 3 | 1 | 5 |
| Valine, leucine and isoleucine degradation | 2 | 1 | 2 | 5 |
| Aldosterone synthesis and secretion | 2 | 1 | 0.5 | 3.5 |
| Proximal tubule bicarbonate reclamation | 1 | 1 | 1.5 | 3.5 |

Supplementary Table 5. References for the association of pathways with symptoms

| Pathways | References |
| --- | --- |
| Bile secretion | PMID: 40052709, PMID: 40045464, PMID: 39700534, PMID: 39032413, PMID: 39719433, PMID: 39594419 |
| Cholesterol metabolism | PMID: 39780051, PMID:40100571, PMID:39884631, PMID: 39954742 |
| Purine metabolism | PMID: 39952622, PMID:39919330 |
| Arachidonic acid metabolism | PMID: 39978863, PMID: 30735766; PMID: 28797264, PMID: 26067486 |
| Protein digestion and absorption | PMID: 40075137 |
| cAMP signaling pathway | PMID: 27035868, PMID: 38218008, PMID: 39448908, PMID: 38113621 |
| Fc gamma R-mediated phagocytosis | PMID: 37867028 |
| Glycerophospholipid metabolism | PMID: 39842075, PMID: 39987603, PMID: 40073453 |
| Mineral absorption | PMID: 39708312 |
| mTOR signaling pathway | PMID: 39976263, PMID: 39708312, PMID: 39793209, PMID: 39995546 |
| Autophagy - animal | PMID: 39002823 |
| Oxytocin signaling pathway | PMID: 39789137, PMID: 39456718, PMID: 34768894 |
| GnRH signaling pathway | PMID: 34176242 |
| Necroptosis | PMID: 35614364, PMID: 37524622 |
| Fc epsilon RI signaling pathway | PMID: 34611316 |
| Valine, leucine and isoleucine degradation | PMID: 39075896, PMID: 40058936, PMID: 39455059 |
| Aldosterone synthesis and secretion | PMID: 39826326 |
| Proximal tubule bicarbonate reclamation | PMID: 35525634 |
